# Thyrotropin-Releasing Hormone (TRH) Promotes Wound Re-Epithelialisation in Frog and Human Skin

**DOI:** 10.1371/journal.pone.0073596

**Published:** 2013-09-02

**Authors:** Natalia T. Meier, Iain S. Haslam, David M. Pattwell, Guo-You Zhang, Vladimir Emelianov, Roberto Paredes, Sebastian Debus, Matthias Augustin, Wolfgang Funk, Enrique Amaya, Jennifer E. Kloepper, Matthew J. Hardman, Ralf Paus

**Affiliations:** 1 Department of Dermatology, University of Luebeck, Luebeck, Germany; 2 Department of Pathology, University of Luebeck, Luebeck, Germany; 3 The Dermatology Centre, Salford Royal NHS Foundation Trust and Institute of Inflammation and Repair, School of Translational Medicine, University of Manchester, Manchester, United Kingdom; 4 Department of Hand and Plastic Surgery, the Second Affiliated Hospital of Wenzhou Medical College, Wenzhou, Zhejiang Province, China; 5 The Healing Foundation Centre, Faculty of Life Sciences, University of Manchester, Manchester, United Kingdom; 6 Department of Vascular Surgery, University Hospital Hamburg-Eppendorf, Hamburg, Germany; 7 Center for Dermatological Research, University Hospital Hamburg-Eppendorf, Hamburg, Germany; 8 Clinic Dr. Koslowski, Munich, Germany; Muséum National d’Histoire Naturelle, France

## Abstract

There remains a critical need for new therapeutics that promote wound healing in patients suffering from chronic skin wounds. This is, in part, due to a shortage of simple, physiologically and clinically relevant test systems for investigating candidate agents. The skin of amphibians possesses a remarkable regenerative capacity, which remains insufficiently explored for clinical purposes. Combining comparative biology with a translational medicine approach, we report the development and application of a simple *ex vivo* frog (*Xenopus tropicalis*) skin organ culture system that permits exploration of the effects of amphibian skin-derived agents on re-epithelialisation in both frog and human skin. Using this amphibian model, we identify thyrotropin-releasing hormone (TRH) as a novel stimulant of epidermal regeneration. Moving to a complementary human *ex vivo* wounded skin assay, we demonstrate that the effects of TRH are conserved across the amphibian-mammalian divide: TRH stimulates wound closure and formation of neo-epidermis in organ-cultured human skin, accompanied by increased keratinocyte proliferation and wound healing-associated differentiation (cytokeratin 6 expression). Thus, TRH represents a novel, clinically relevant neuroendocrine wound repair promoter that deserves further exploration. These complementary frog and human skin *ex vivo* assays encourage a comparative biology approach in future wound healing research so as to facilitate the rapid identification and preclinical testing of novel, evolutionarily conserved, and clinically relevant wound healing promoters.

## Introduction

Non-healing skin ulcers represent an area of major clinical challenge [1]–[4] urgently requiring more effective and safe treatments. Specifically, topical agents are required that can induce and/or promote wound re-epithelialisation, a critical limiting factor in chronic wounds [1], [5]–[7]. While keratinocyte mitogens, such as cytokines and growth factors, have been identified in animal studies [8], [9] few have made it into the clinical trial stage and even fewer into daily clinical practice. In seeking additional potential wound-healing promoters, we have therefore moved outside the traditional animal models of cutaneous wound repair (mouse, rabbit, pig) and have employed the consecutive organ culture of adult frog and human skin as a cross-species wound healing research system that facilitates the rapid identification and preclinical testing of novel, evolutionarily conserved, and clinically relevant wound healing promoters.

**Figure 1:**
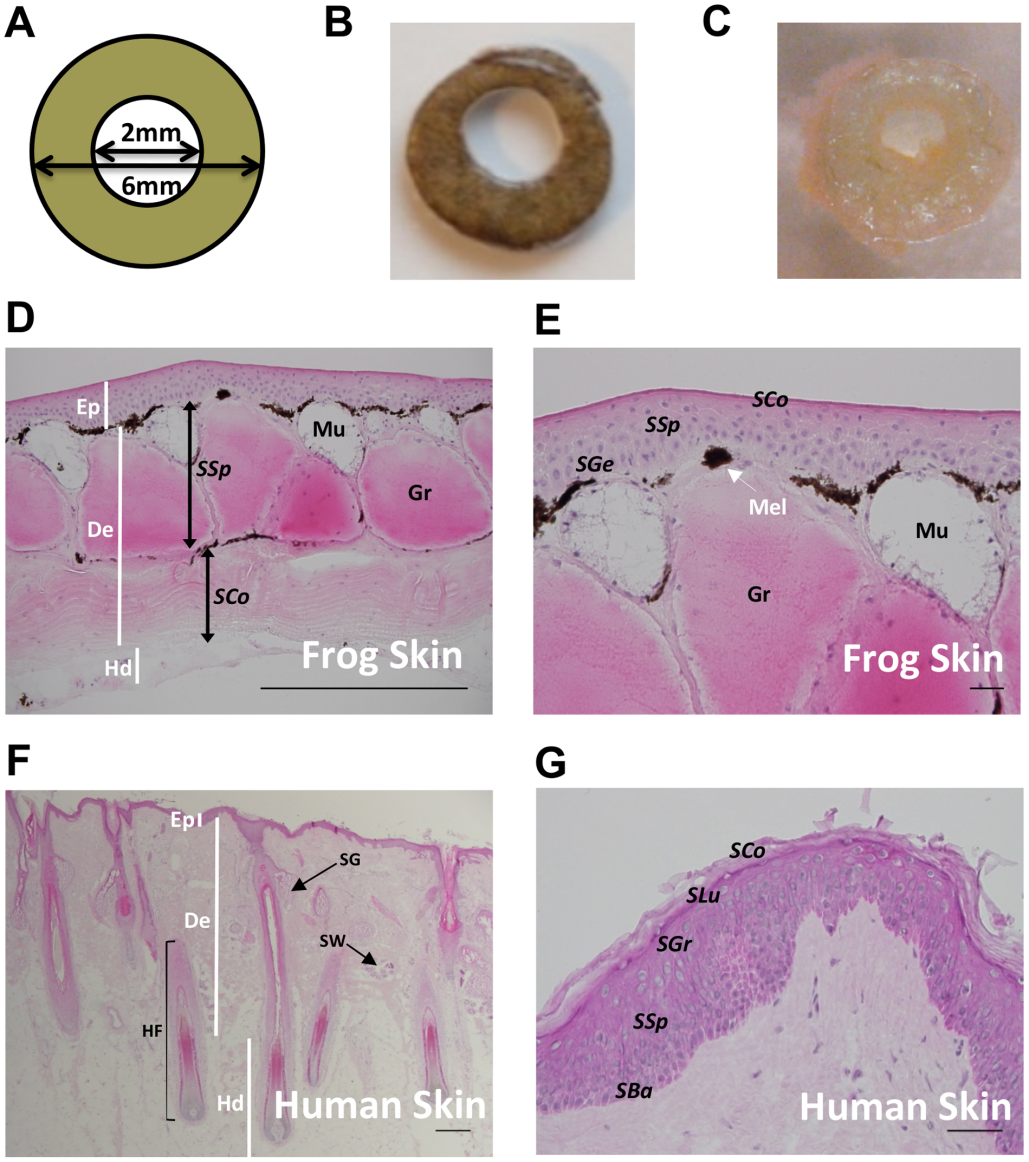
Wound healing assay design and morphology of *Xenopus tropicalis* and human skin. (**A**) Diagram of the ‘punch within a punch’ biopsy injury inflicted on both *X. tropicalis* and human skin (not to scale). (**B**) *X. tropicalis* punch-in-a-punch biopsy. (**C**) Human skin punch-in-a-punch biopsy. Full thickness *X. tropicalis* (**D**) and human skin (**F**) with white lines demarcating epidermis (Ep), dermis (De) and hypodermis/subcutis (Hd). Black arrows in (**D**) indicate the margins of the *stratum spongiosum (SSp) stratum compactum (SCo)*. A smaller mucous (Mu) and larger granular/poison (Gr) gland are indicated (**D**). Human sebaceous glands (SG) and sweat glands (SW) and hair follicle (HF) are indicated (**F**). Higher magnification images (**E & G**) display the epidermis, specifically indicating *Stratum germinativum (SGe)*, *Stratum spinosum (SSp)* and *Stratum corneum (SCo)* of frog epidermis (**C**) and *Stratum basale (Sba) stratum spinosum (SSp), Stratum granulosum (SGr), Stratum Lucidum (SLu) and Stratum corneum (SCo)* of human epidermis (**G**). A white arrow shows the location of a melanocyte (Mel) at the dermal-epidermal junction. (**E**). Scale bars in D and F = 300 µm and in E and G = 50 µm.

Amphibians and lizards show highly efficient wound repair [10]–[14] up to the point of regeneration of an entire limb or tail in juvenile animals [15]–[17]. Frog and human skin share the same evolutionary ancestry and basic architecture and although frog skin dermis differs from its mammalian counterpart with respect to its gland-rich *stratum spinosum*, its collagen-rich *stratum compactum* closely resembles mammalian dermis [16], [17] (see also Figure 1). Assuming that the broad mechanisms of wound healing are conserved between human and frog skin, we suggest that the systematic study of regenerative amphibian healing may reveal important pointers as to how human skin wound healing may be promoted. We have been drawn to those conserved neuroendocrine molecules that are abundant in frog skin. Specifically we have investigated in this study the potential role of thyrotropin-releasing hormone (TRH) in cutaneous wound repair in the frog.

TRH, the chief hypothalamic regulator of thyroid hormone production [18], [19], is abundant in adult frog skin (*Xenopus laevis* skin contains up to 15 µg/g of TRH) [20], [21]. TRH is also present in human skin [22] where it acts as a potent stimulator of human hair growth, follicle keratinocyte proliferation and mitochondrial energy metabolism *in situ* [22]–[24]. Since there are multiple biological parallels between hair growth and wound healing [25]–[28], we hypothesised that, a) TRH may also function as a novel promoter of frog skin wound healing, and b) this role may be conserved in human skin healing as TRH is identical in frogs and humans [31].

To investigate this hypothesis, we have developed a reproducible *ex vivo* assay for amphibian skin wound healing that, when paired with a complementary human *ex vivo* assay, facilitates a comparative biology approach to vertebrate wound healing research across the amphibian-human species divide. We believe that this cross-species, comparative approach provides an important technical advance over previously reported *ex vivo* wound healing models [29], [30]. Drawing upon the long tradition of amphibian skin organ culture [32]–[35], we identified the skin of adult *X. tropicalis* frogs as being optimally suited to organ culture. In contrast to *X. laevis*, skin explants from *X tropicalis* displayed minimal shrinkage during culture, were less prone to infection, and can be incubated at higher temperatures (25°C). A basic organ culture design [36] previously optimised for human skin organ culture [37] was chosen to maximise the cross-species comparative potential of this complimentary assay system. In parallel, we developed a complimentary *ex vivo* human skin wound healing assay based on the same design [36]. In contrast to a recently published model in which partial thickness human skin and superficial incisional wounds were studied in serum-supplemented DMEM medium [29], our assay employs full-thickness human and frog skin, injured with excisional punch wounds and a *serum-free* medium that is essentially identical between the frog and human wounded skin assays (corrected only for osmolality).

By culturing both *X. tropicalis* and human skin explants in the absence of serum, effective wound healing was impaired (as previously described) [38], allowing for the effects of putative wound healing stimulatory agents to be investigated. In both systems, the addition of serum (frog or human, respectively) or estrogen, both well-established wound-healing promoters in rodents and man [38], [39], were employed as positive controls. Use of essentially the same medium for both the frog and human skin wound healing assay further facilitates the cross-species comparison of test agents.

Using this comparative biology approach, we demonstrate that TRH stimulates re-epithelialisation in both adult frog and adult human skin, thus identifying this endogenous peptide neurohormone of frog and human skin as a novel, clinically relevant and evolutionarily conserved promoter of cutaneous wound healing across distant vertebrate species.

## Materials and Methods

### Ethics Statement

All experiments were performed according to the Helsinki guidelines and following approval by the Institutional Research Ethics Committee of the University of Lübeck. All patients provided both written and verbal informed consent. Consent forms (Version number 06–109) were approved for use by the Institutional Research Ethics Committee of the University of Lübeck. Tissue harvesting from sacrificed animals was governed by UK Home Office guidelines. Tissue harvesting from Schedule 1 sacrificed animals was covered by guidelines laid down by the University of Manchester Animal Welfare Centre following the UK Home Office Animal Licence Act, 1997.

### Frog Skin Wound Healing Assay

Male and female adult *X. tropicalis* frogs (approximately 2 years old) were sacrificed. Dorsal skin was decontaminated using a non-alcoholic disinfectant (Octenisept® spray; Schuelke, Germany), and removed by incising through the middle of the lateral line system with surgical scissors (World Precision Instruments, Hitchin, UK). Skin was placed into 50 ml of Williams E (WE) medium in a 120 mm tissue culture dish, containing 200 IU/mL penicillin/20 µg/mL streptomycin, for two hours at 2% CO_2_, 25°C. Skin sheets were placed dorsal side up on a flat, sterile surface, striving for minimal skin trauma so as to reduce frog skin mucous production. Circular wounds were inflicted: First, 6 mm punches were cut into skin using a biopsy punch (PFM, Köln, Germany). Smaller (2 mm) biopsy punches were then used to create “punch-in-a-punch” skin wounds (Figure 1). These were individually placed, with the epidermis facing up, in 24-well plates and cultured in serum-free WE medium diluted 1∶1 in sterile distilled water, supplemented with 10 µg/mL insulin, 10 ng/mL hydrocortisone, 2 mM L-glutamine and 100 IU/mL penicillin/10 µg/mL streptomycin**.** Frog skin explants were cultured in the absence (negative control) or presence of 5% frog serum isolated from *X. laevis* adult female frogs ( =  positive control). In addition, 17β-estradiol was investigated as an additional positive control substance for the promotion of wound healing [39]. Biopsies were harvested at day 0, day 1, day 3 and day 7 with media changes every 48 hours.

### Assessment of Re-epithelialisation, and Immuno/Histomorphometric Analysis of Proliferation and Apoptosis

The progression of re-epithelialisation within the interior 2 mm wound circle was assessed microscopically using a Leica DMIL inverted microscope (Leica GmbH, Wetzlar, Germany) fitted with a Panasonic DMC-L10 (Panasonic, Osaka, Japan) SLR camera. The degree of re-epithelialisation within the wound area was calculated as previously described [40].

For paraffin sections, skin biopsies were fixed in zinc fixative before paraffin embedding. 7 µm thick paraffin sections were cut from each specimen, and stained with haematoxylin and eosin (H&E). For frozen sections, skin fragments were embedded in Shandon Cryomatrix (Thermo Fisher Scientific; Waltham, MA, U.S.A.) and snap frozen in liquid N_2_ before 7 µm thick cryosections were prepared for analyses.

To assess proliferation, immunohistochemical analysis of the mitosis marker phospho-histone H3 (PH3) with a specific antibody (Millipore, CA, U.S.A.; 1∶1000) was performed on acetone-fixed cryosections. Following overnight incubation with the primary antibody, secondary immunofluorescence was detected using the Alexa Fluor 488 probe (Molecular Probes, Eugene, OR). Nuclei were visualized with 4, 6-diamino-2-phenylindole (DAPI; 1 µg/mL). As an additional assessment of proliferation in frog skin, Weigerts iron-haematoxylin histochemistry was performed in order to identify mitotic figures. Briefly, slides were deparaffinised and placed in freshly mixed Alcoholic Haematoxylin and Acid Aqueous Ferric Chloride solution for two hours. They were then differentiated in 0.5% acid alcohol before ‘blueing’ in running tap water. Finally, slides were counterstained with Eosin, rinsed in running tap water and mounted.

Apoptotic cells were detected using the TUNEL method (Apoptag ® fluorescein detection kit; Millipore, Watford, UK). Images were captured using a Keyence Biozero-8000 Microscope (Keyence Corporation, Osaka, Japan). Analysis of carefully defined reference areas was performed as previously described [41]–[44] using NIH ImageJ software (National Institutes of Health, Bethesda, MD, U.S.A.).

### Estradiol and TRH ELISA

The estradiol concentration in frog serum was determined using the enzyme immunoassay kit Estradiol EIA (Cayman Chemical, Ann Arbor, USA) according to manufacturer’s guidelines. The TRH ELISA kit (Uscn Life Science Inc, Wuhan, China) was used, according to manufacturer’s guidelines, for determining TRH frog serum concentrations.

### Human Wound Healing Organ Culture Assay

Excess scalp and body skin from 4 women (52, 60, 66 and 67 years of age) undergoing elective cosmetic surgery was obtained after informed consent and ethics approval (University of Lübeck, Germany). The “punch-in-a-punch” design [36] was employed (2 mm and 4 mm circular wounds, Figure 1), the notable difference being that hair-bearing, full-thickness (i.e. including subcutaneous fat) adult human skin was used, with cultures performed using *serum-free* WE medium optimized for human hair follicle organ culture [45]. This culture medium is essentially identical to that used in the *X. tropicalis* cultures described above (with the exception of a 1∶1 dilution with water in the frog assay) allowing for direct comparisons in wound-healing responses. As with the comparative frog skin cultures, 17β-estradiol was also investigated as a positive control for the promotion of wound healing [39].

### (Immuno-)histological Analyses and Quantitative Immunohistomorphometry

Human skin biopsies were embedded in Shandon Cryomatrix before cutting longitudinal 6 µm cryosections for further analyses. Routine histology was performed by staining with Mayer’s Haematoxylin (Merck, Darmstadt, Germany) and 0.1% Eosin E (Sigma-Aldrich, St Louis, MO, U.S.A.). Proliferating and apoptotic cells were analysed by quantitative Ki-67/TUNEL immunohistomorphometry, using ImageJ software as described [41]. Nuclei were visualized with 4,6-diamino-2-phenylindole (DAPI). Sections were stained for α-cytokeratin 6 (CK6) using the primary antibody (Progen, Heidelberg, Germany) diluted at 1∶10. Detection of CK6 was performed with goat-anti-mouse-IgG-FITC/−Rhodamine (JIR, Westgrove, PA; USA; 1∶200). Involucrin detection was preformed as previously described [42]. Images were captured using a Keyence Biozero-8000 Microscope (Keyence Corporation, Osaka, Japan). Quantitative immunohistomorphometry of carefully defined reference areas in test and control sections was performed as described [40]–[44].

### Statistical Analysis

For human and frog skin culture, statistical analysis was carried out using one-way analysis of variance (ANOVA) with Bonferroni’s post-tests. *P* values of <0.05 were regarded as significant.

## Results

### Frog Serum and Estrogen Enhance Re-epithelialisation and Proliferation of ex vivo Wounded Adult *X. tropicalis* Skin

In a series of preparatory experiments we had established that full-thickness (∼150 µm) skin of adult *X. tropicalis* provides a viable tissue source for skin organ culture and is superior in this respect to *X. laevis*, whose skin is more fragile and requires lower temperature culture conditions (data not shown). Six mm punch biopsies of adult frog skin were wounded via a central 2 mm biopsy (“punch-in-a-punch” design; Figure 1) and cultured in a modified serum-free growth medium previously optimised for human skin and hair follicle culture. The quick and easily reproducible measurement parameter of absolute wound closure was used to planimetrically quantify re-epithelialisation.

**Figure 2:**
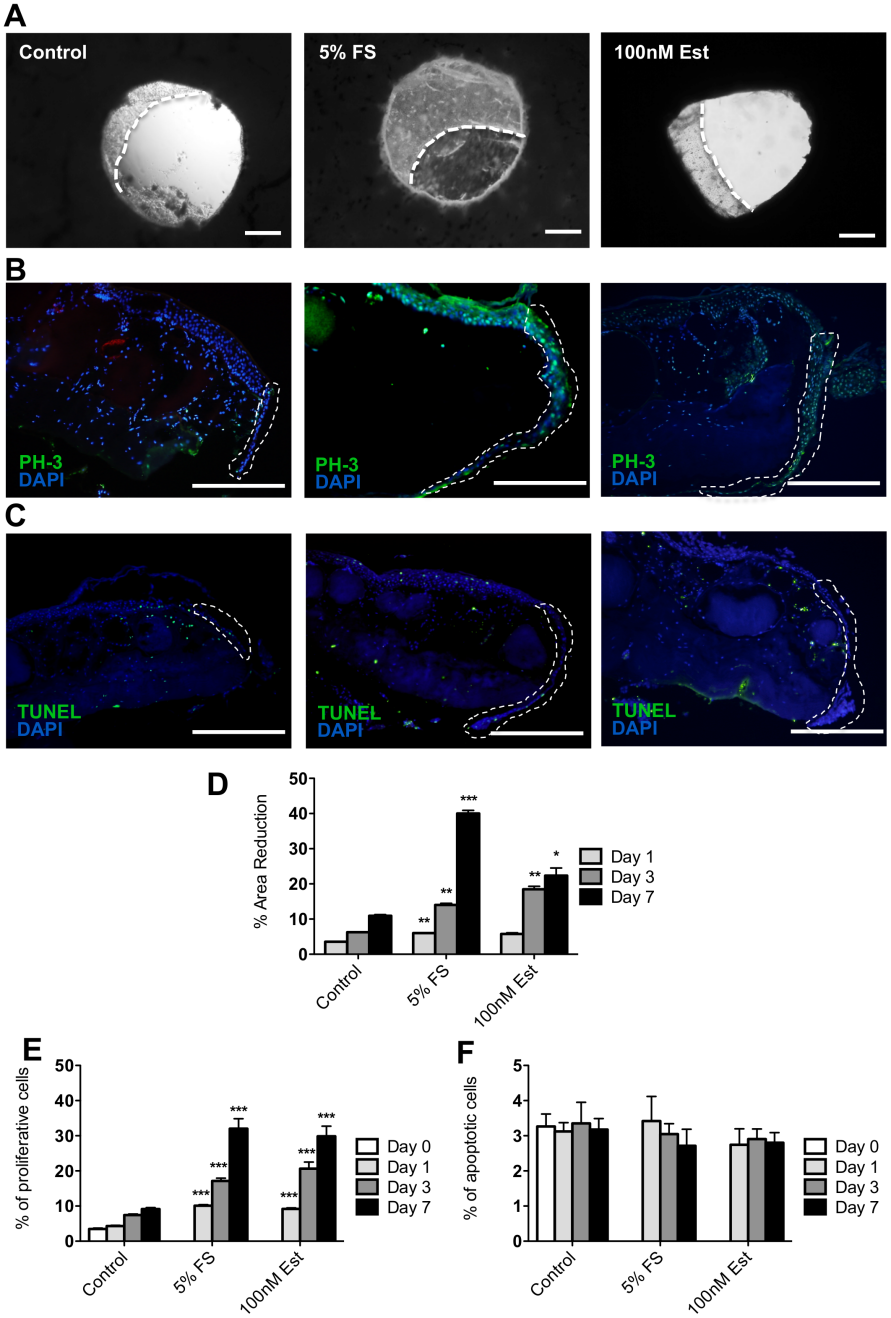
Frog serum and estrogen promote re-epithelialisation and proliferation in *Xenopus tropicalis* skin. (**A**) Representative images of re-epithelialisation sheet in control, 5% frog serum and 100 nM 17β-estradiol (Est)-treated punch wounds at day 7 in culture (scale bars: 0.5 mm). White-hatched lines indicate the leading edge of new epithelial sheets. (**B**) Representative images of PH3-positive cells in control, 5% frog serum and 100 nM 17β-estradiol-treated punch wounds at day 7 in culture. The white-hatched line demarcates new epithelial tongue. (**C**) Representative images of TUNEL-positive cells in control, 5% frog serum and 100 nM 17β-estradiol-treated punch wounds at day 7 in culture. The white-hatched line demarcates new epithelial tongue. Scale bars in (**B**) and (**C**) are 100 µm. (**D**) The graph shows the percentage reduction in wound-area of *Xenopus tropicalis* punch wounds in control, 5% frog serum (FS) and 100 nM 17β-estradiol-treated skin. (**E**) Percentage of proliferative (PH3-positive) cells present in the new epithelial tongue during re-epithelialisation. (**F**) Percentage of apoptotic (TUNEL-positive) cells present in the new epithelial tongue during re-epithelialisation. Data are mean ± SEM of 4–5 frogs (2 male and 3 female). Significance relative to control data at the same time-point denoted by *P<0.05, **P<0.01, ***P<0.001.

Over 7 days frog skin organ culture in minimal media we observed a small (∼10%) reduction in the wound area (Figure 2A,D). Since serum has long been appreciated as a wound-healing promoter in other species [46]–[48], we tested the effect of normal frog serum supplementation. Healing was dramatically accelerated following treatment with 5% adult female frog (*X. laevis*) serum (mean 40% wound closure after seven days; Figure 2A,D). In addition treatment with 17β-estradiol, a widely accepted wound healing promoter [39], significantly enhanced wound closure compared to controls (Figure 2A,D). Re-epithelialisation appeared to progress unevenly along the wound edge of the inner punch biopsy (Figure 2A). Although one might expect this process to proceed evenly across the circular wound, it appears that a focal area is established, from which migration and proliferation expands rapidly until the advancing sheet covers the entire wound area.

To investigate potential wound-healing stimulatory components in isolated frog serum, we assessed the concentrations of both 17β-estradiol and TRH by ELISA. Results indicate the serum 17β-estradiol levels were 29.7 nM (8.1 ng/mL). Serum TRH levels were below the limit of detection.

Keratinocyte proliferation plays an important role in mammalian re-epithelialisation. In unwounded frog skin (day 0) proliferation, measured by the proportion of PH3 positive (mitotic) cells, was low and similar to levels seen in unwounded mammalian skin (around 3%; Figure 2E). Over the seven days following wounding and skin organ culture a modest increase in proliferation was observed in minimal media (Figure 2B,E). However, the addition of either 5% frog serum or 100 nM 17β-estradiol led to a rapid and substantial increase in proliferative, PH3 positive cells (Figure 2B,E). Finally, TUNEL assays were performed to establish the contribution of apoptosis to the observed wound closure. The number of apoptotic cells in the wound edge and neo-epidermis was low (∼3%) and essentially unaltered in the presence of frog serum or 17β-estradiol (Figure 2C,F).

### TRH Promotes Re-epithelialisation and Proliferation in Frog Skin ex vivo Wounds

The neuropeptide TRH is prominently and constitutively expressed in frog skin [20] and acts as a potent stimulator of human hair growth [22] and mitochondrial energy metabolism of human epidermis and hair follicles [23], [24]. We therefore examined the effects of exogenous TRH application on wounded *ex vivo* cultured frog skin. The concentrations of TRH investigated were selected to be comparable with those concentrations previously shown to stimulate hair growth (10 nM) [22] and the high concentrations found in the skin of certain frog species such as *X. laevis* (10 µM) [20], [21].

**Figure 3:**
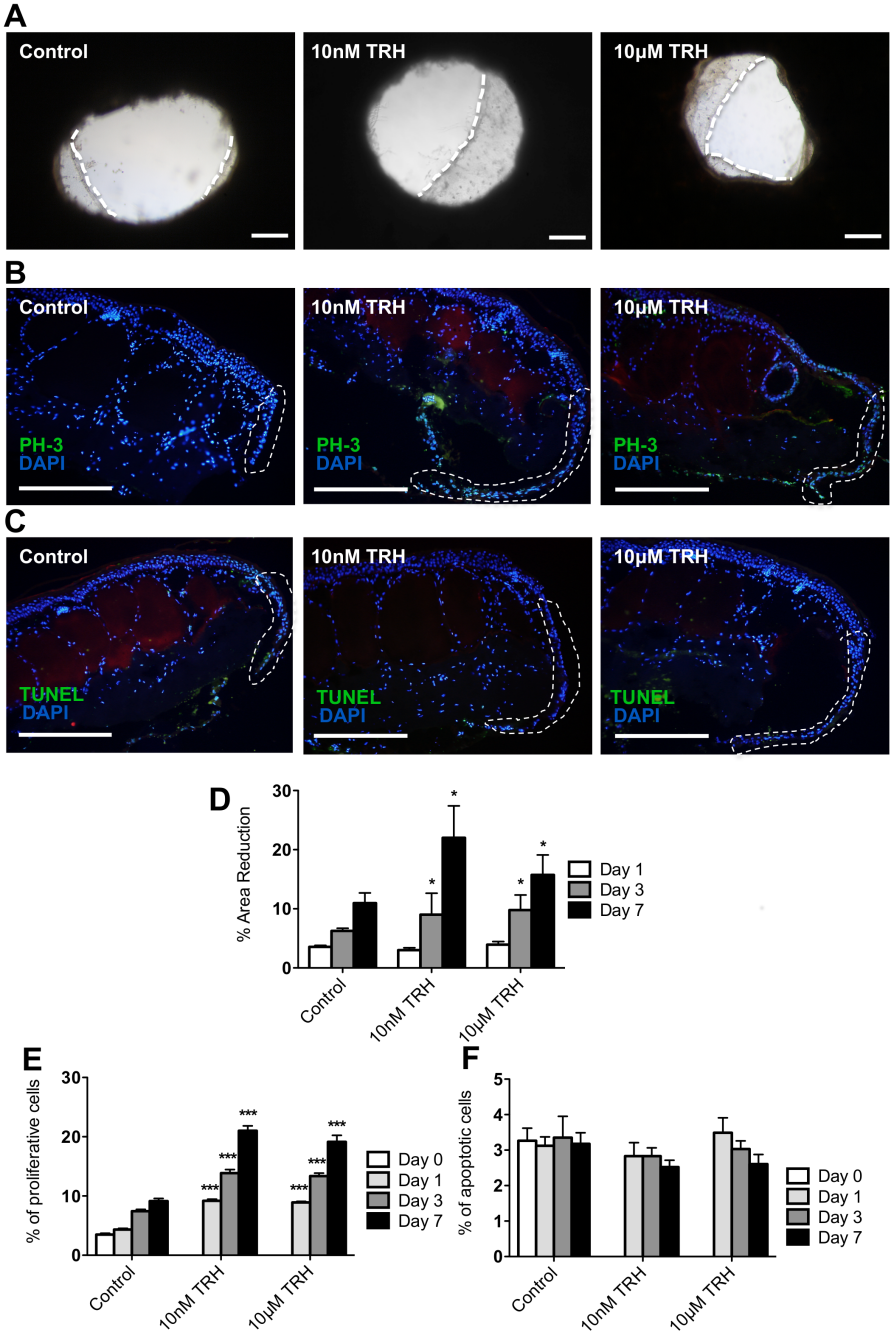
*Xenopus tropicalis* wound closure: promotion of re-epithelialisation and proliferation by TRH. (**A**) Representative images of the re-epithelialisation sheet in control, 10 nM and 10 µM TRH-treated punch wounds at day 7 in culture (scale bars: 0.5 mm). White-hatched lines indicate the leading edge of new epithelial sheets. (**B**) Representative images of PH3-positive cells in control, 10 nM and 10 µM TRH-treated punch wounds at day 7 in culture. The white-hatched line demarcates new epithelial tongue. (**C**) Representative images of TUNEL-positive cells in control, 10 nM and 10 µM TRH-treated punch wounds at day 7 in culture. The white-hatched line demarcates new epithelial tongue. Scale bars in (**B**) and (**C**) are 100 µm. (**D**) The graph shows the percentage reduction in wound-area of *X. tropicalis* punch wounds in control, 10 nM and 10 µM TRH-treated skin. (**E**) Percentage of proliferative (PH3-positive) cells present in the new epithelial tongue during re-epithelialisation. (**F**) Percentage of apoptotic (TUNEL-positive) cells present in the new epithelial tongue during re-epithelialisation. Data are mean ± SEM of 16 frogs (8 male and 8 female). Significance relative to control data at the same time-point denoted by *P<0.05, ***P<0.001.

**Figure 4:**
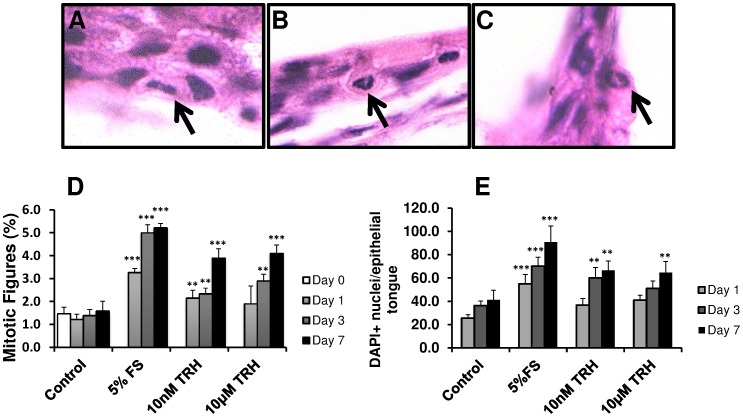
*Xenopus tropicalis* mitosis: TRH increases epidermal mitosis. (**A–C**) Representative images of Weigert’s stained *X. tropicalis* epidermis with black arrows indicating mitotic cells. (**D**) The graph shows the percentage of mitotic figures identified by Weigert’s staining in the *X. tropicalis* epidermis. 200 nuclei were analysed per skin section, with 3 sections per animal counted. (**E**) The graph displays the total number of DAPI+ nuclei in the new epithelial tongues. Data are mean ± SEM of 4 frogs (2 male and 2 female). Significance relative to control data at the same time-point denoted by **P<0.01, ***P<0.001.

At the gross morphological level, both 10 nM (equivalent to 3.6 ng/ml) and 10****µM (equivalent to 3.6 µg/ml) TRH significantly stimulated wound closure (Figure 3A,D). PH3 analysis revealed a strong stimulatory effect of TRH on keratinocyte proliferation at all time-points assessed (Figure 3B,E). Increased proliferation was also indicated by Weigert’s iron hematoxylin histochemistry, by which an increased number of mitotic epidermal cells were identified after TRH treatment, compared to vehicle controls (Figure 4). As with frog serum, TRH treatment did not significantly change the number of TUNEL+ apoptotic cells (Figure 3C,F). These findings provide the first evidence that this tripeptide neurohormone is a potent stimulator of frog skin epithelial repair.

There were no differences observed in dermal proliferation following 5% FS, 10 nM or 10****µM TRH exposure. Figure S1 indicates the percentage of PH3+ cells in the dermis. This result was confirmed by Weigert’s iron hematoxylin histochemistry, which demonstrated no differences in the number of visible mitotic figures in the dermis (Figure S1).

### Human Serum and Estrogen Stimulate Human Skin Re-epithelialisation and Proliferation ex vivo

**Figure 5:**
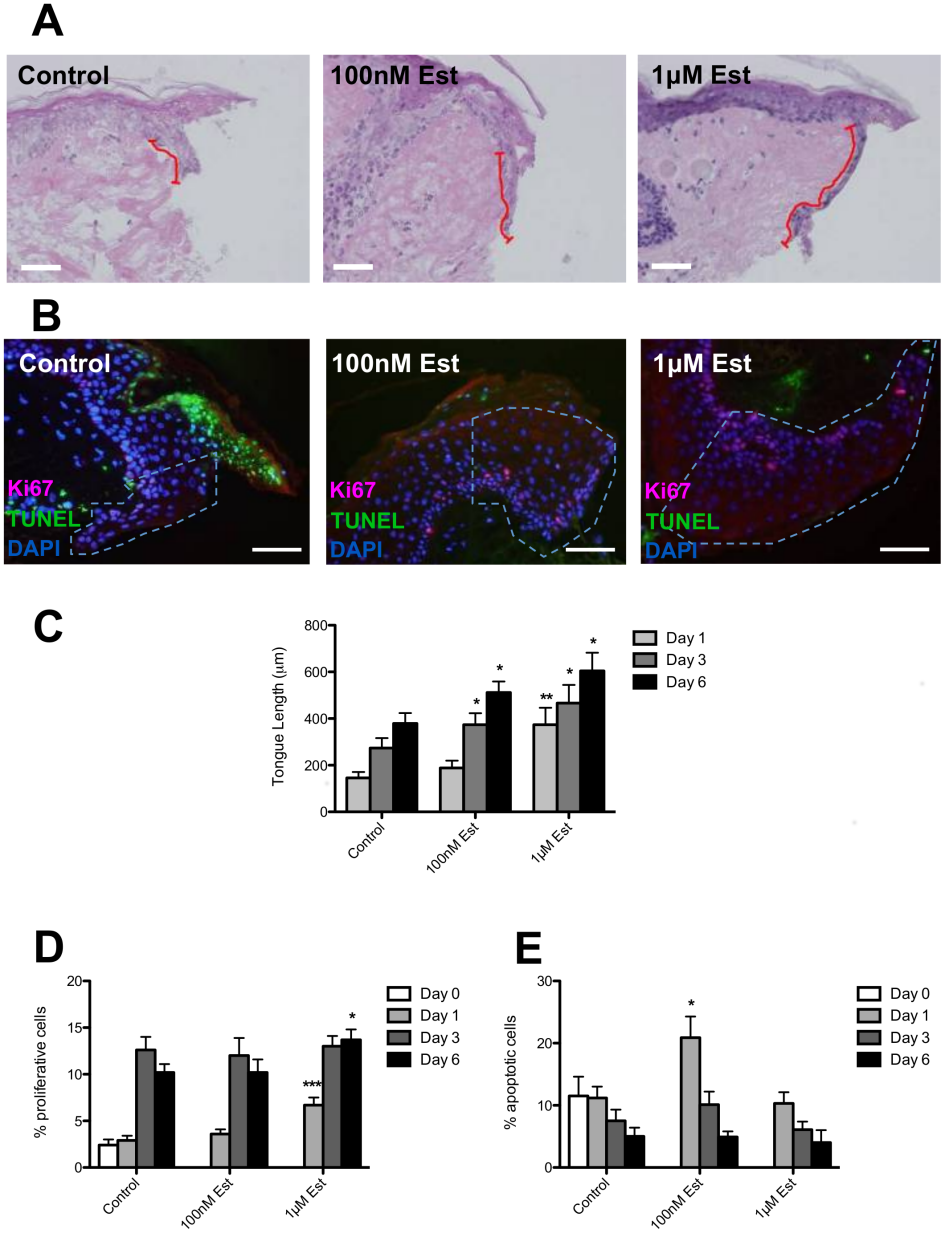
Increased re-epithelialisation and proliferation in human skin following 17β-estradiol treatment. (**A**) Representative H&E stained sections of human skin punch-wounds following 6 days culture with either vehicle control, 100 nM or 1 µM 17β-estradiol (Est). New epithelial tongue indicated by red lines, scale bar represents 50 µm. (**B**) Representative images of Ki-67-TUNEL double-stained sections of human skin punch wounds following 6 days culture with either vehicle control, 100 nM or 1 µM 17β-estradiol. The white-hatched line demarcates new epithelial tongue. (**C**) The graph displays length measurements of the new epithelial tongue (as demarcated by the red lines in **A**) as analysed from H&E stained human skin sections following treatment with vehicle control, 100 nM and 1 µM 17β-estradiol. (**D**) Percentage of proliferative (Ki67-positive) cells in the new epithelial tongue of vehicle control, 100 nM and 1 µM 17β-estradiol-treated human skin wounds. (**E**) Percentage of apoptotic (TUNEL-positive) cells in the new epithelial tongue of vehicle control, 100 nM and 1 µM 17β estradiol-treated human skin wounds. Data are mean ± SEM of 4 female donors. Significance relative to control data denoted by *P<0.05, **P<0.01, ***P<0.001.

To facilitate cross-species comparison we have established a directly comparable human *ex vivo* wound healing assay. The epithelial wound healing response comprises a carefully regulated combination of keratinocyte proliferation and migration. Thus, we explored the contribution of proliferation versus cell migration to epithelial tongue formation in our *ex vivo* human wound model. Wounded adult human skin displayed prominent epiboly [49] within 24 h of wounding, with a compact rim of epidermal keratinocytes forming an “epithelial tongue” that covered the exposed dermis at the inner wound edge. As an initial approximation of re-epithelialisation we measured the overall length of newly formed inner and outer epithelial tongues. As with the frog skin organ cultures, the known wound-healing promoter 17β-estradiol [39] significantly stimulated epithelial migration, as documented by epithelial tongue length measurements, at days 3 and 6 post-wounding at low dose (100 nM) and at days 1, 3 and 6 at high dose (1 µM), compared to controls (Figure 5A,C).

Within the “epithelial tongues”, 1 day after standardized punch wounding, there were relatively few Ki67+ (proliferative) cells present in the control wounds (∼3%) (Figure 5B,D). However, by day 6 approximately 10% of the cells in the newly formed outer and inner epithelial tongues of control wounds were Ki67+ (Figure 5B,D). This exactly mirrors the *in vivo* hyperproliferation response where a proliferative burst within the epithelial wound edge occurs >24 h post wounding. In our *ex vivo* model we observed increased epithelial tongue proliferation following 17β-estradiol treatment at the higher dose (1 µM) only (Figure 5B,D). This suggests that the impact of estrogen at the lower dose is primarily on epithelial migration, in line with published observations *in vivo* [39]. Again, there was little difference in TUNEL+ (apoptotic) cells in the epithelial tongues following 17β-estradiol treatment compared to vehicle control skin (Figure 5B,E).

### Human Skin Re-epithelialisation ex vivo Involves Sequential Up-regulation of Distinct Differentiation Programmes: Cytokeratin 6 and Involucrin Expression

**Figure 6:**
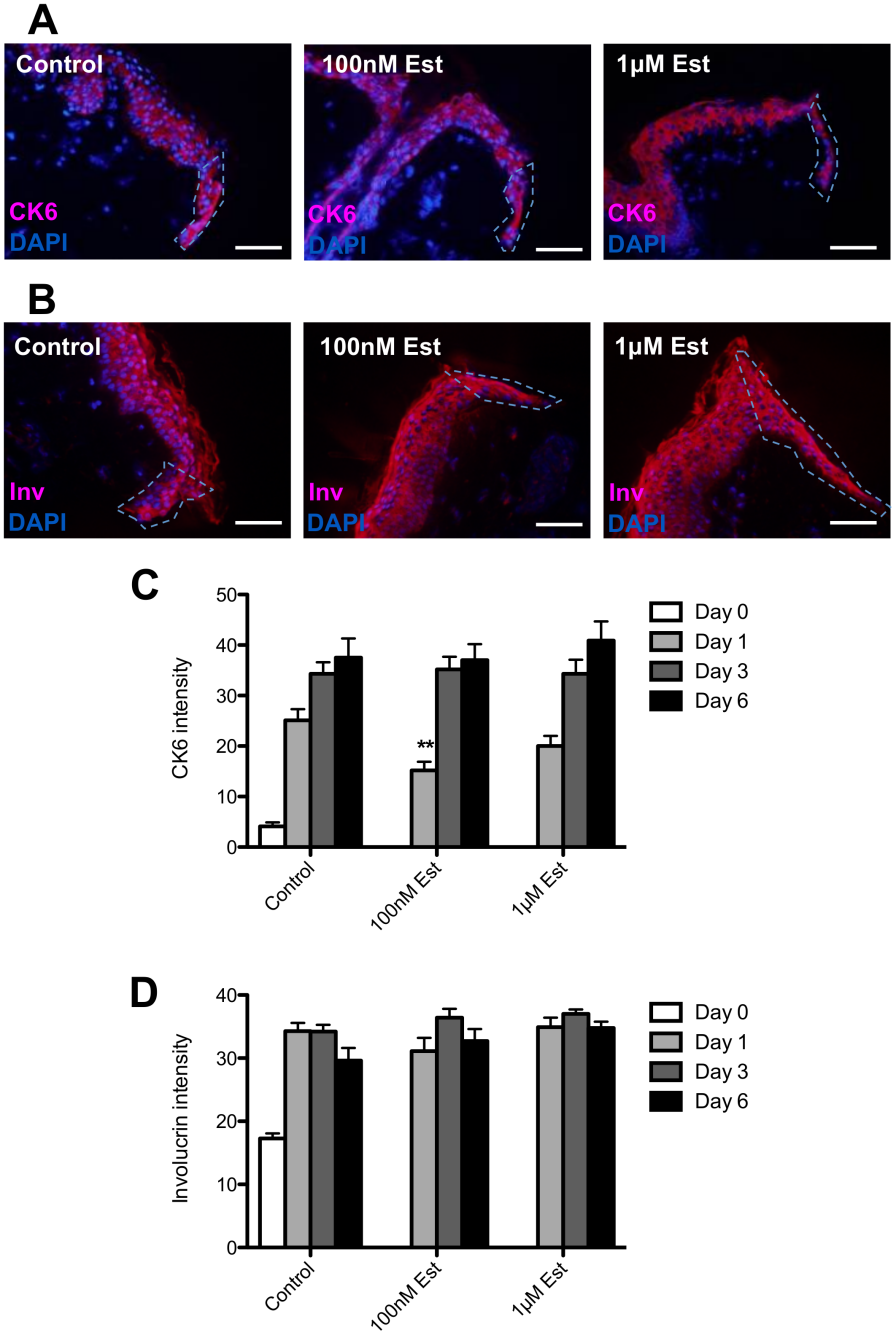
Cytokeratin 6 and Involucrin expression in human skin punch wounds following 17β-estradiol treatment. Representative images indicating cytokeratin 6 (CK6) expression in control, 100 nM and 1 µM 17β-estradiol (Est)-treated human skin punch wound sections, 6 days post-wounding. The white-hatched line demarcates new epithelial tongue. (**B**) Representative images indicating involucrin (Inv) expression in control, 100 nM and 1 µM 17β-estradiol-treated human skin punch wound sections, 6 days post-wounding. The white-hatched line demarcates new epithelial tongue. (**C**) Quantification of CK6 immunoreactivity in the new epithelial tongues indicated (**A**). (**D**) Quantification of involucrin immunoreactivity in the new epithelial tongues indicated in (**B**). Data are mean ± SEM of 4 female donors. Significance relative to control data denoted by **P<0.01.

We analysed the expression of cytokeratin 6 (CK6), which is found in human inter-follicular epidermis only upon injury and in states of hyperproliferation [50]–[53]. Strong CK6 immunoreactivity was evident one day post-wounding and was maintained at high levels thereafter. Interestingly, epidermal CK6 induction preceded the proliferative peak in control wounds (Figure 6A,C) supporting evidence that CK6 is a sensitive early marker for regenerating normal human skin epithelium *in situ* [29].

17β-estradiol had little effect on CK6 expression, with a small but significant down-regulation only evident in the first day of healing. Immunoreactivity for involucrin, a marker of keratinocyte terminal differentiation, also displayed an early peak at day 1 and was maintained thereafter at high level (Figure 6B,D). Intriguingly, the involucrin staining pattern also showed a shift in its localisation in the epithelial tongue, *i.e.* from its normal expression pattern with maximal intensity in the epidermal granular layer to a more diffuse distribution in all suprabasal layers of the epithelial tongue (Figure 6B). This resembles the ectopic involucrin expression pattern previously reported in psoriasis [54], epidermal tumours [55], [56], and cultured skin substitutes [57].

### TRH Stimulates Human Skin Re-epithelialisation and Proliferation

**Figure 7:**
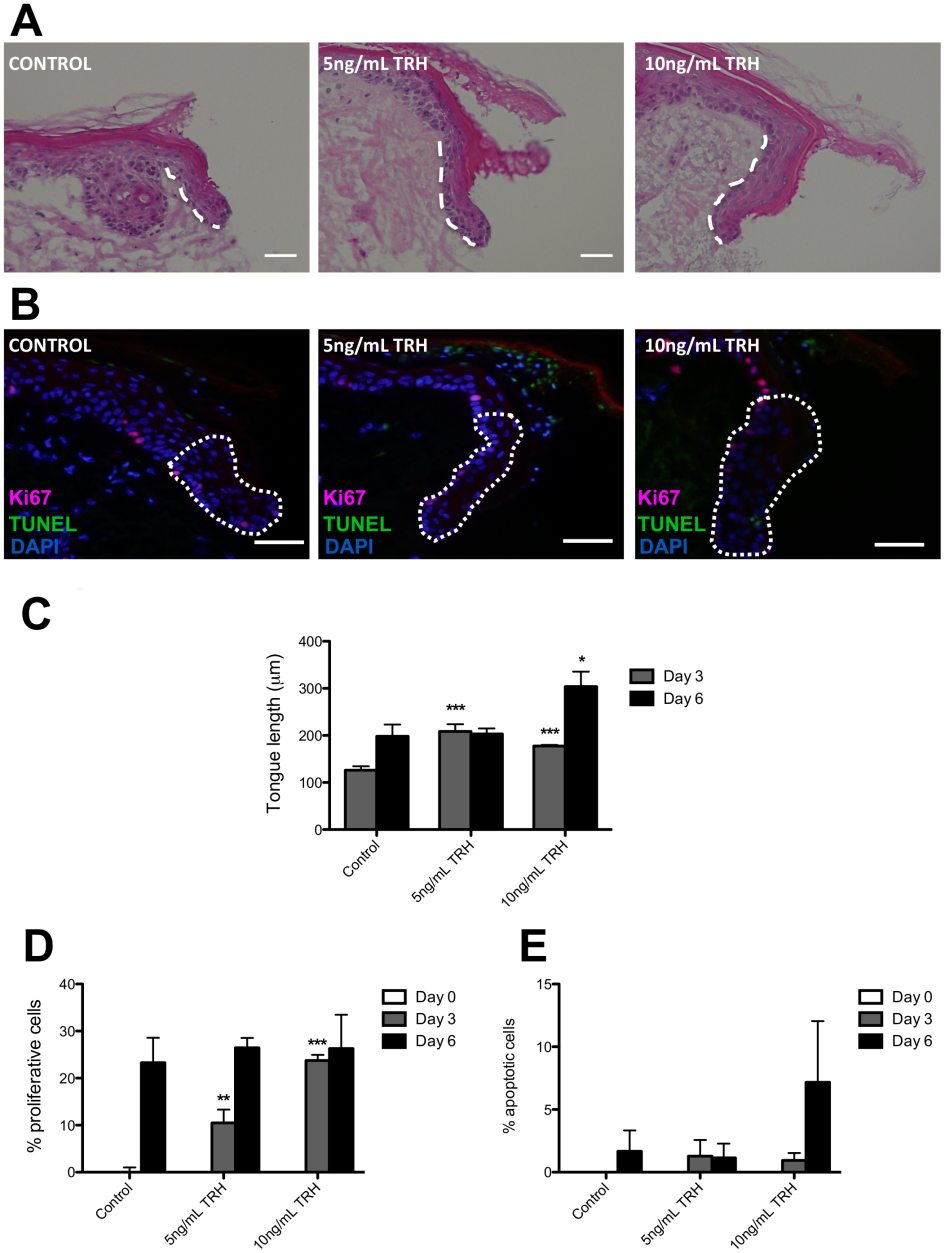
TRH stimulates re-epithelialisation and enhances proliferation in wounded human skin. (**A**) Representative H&E stained sections of human skin punch-wounds following 6 days culture with either vehicle control or 10 ng/mL TRH. New epithelial tongues indicated by the white-hatched lines. Scale bars represent 50 µm. (**B**) Representative images of Ki-67-TUNEL double-stained sections of human skin punch wounds following 6 days culture with either vehicle control or 10 ng/mL TRH. The white-hatched line demarcates new epithelial tongue. (**C**) The graph displays length measurements of the new epithelial tongue (as demarcated by the white-hatched lines in **A**) as analysed from H&E stained human skin sections following treatment with vehicle control, 5 ng/mL and 10 ng/mL TRH. (**D**) Percentage of proliferative (Ki67-positive) cells in the new epithelial tongue of vehicle control, 5 ng/mL and 10 ng/mL TRH-treated human skin wounds. (**E**) Percentage of apoptotic (TUNEL-positive) cells in the new epithelial tongue of vehicle control, 5 ng/mL and 10 ng/mL TRH-treated human skin wounds. Data are mean ± SEM of 4 female donors. Significance relative to control data at the same time-point denoted by *P<0.05, **P<0.01, ***P<0.001.

We next investigated the impact of the intracutaneously generated tripeptide neurohormone, TRH [19], [20]–[22], as a potential wound-healing promoter in human skin. For this, we tested two doses of TRH (5 and 10 ng/ml; 14 and 28 nM, respectively). These concentrations were selected as both had previously been shown to stimulate *ex vivo* human hair growth [22]. In addition they were consistent with the lower of the two TRH doses tested on frog skin organ culture (3.6 ng/ml, equivalent to 10 nM). Both TRH concentrations significantly stimulated epithelial tongue formation in wounded, organ-cultured human skin at day 3 post-wounding (P<0.01) (as quantified by measurement of epithelial tongue length), with maximal effects exerted by 10 ng/ml TRH treatment (Figure 7A,C). Quantitative Ki-67 immunohistomorphometry revealed an accelerated onset of keratinocyte proliferation at day 3 in TRH-treated human epithelial tongues (Figure 7B,D). As in frog skin wounds, TRH treatment did not significantly change intraepithelial apoptosis (TUNEL immunohistomorphometry) (Figure 7B,E). Thus, apoptosis-driven remodelling does not constitute a major part of the wound-healing response in these *ex vivo* skin cultures. These data provide the first evidence that TRH is an evolutionarily conserved stimulator of re-epithelialisation in both adult frog and human skin.

### TRH Up-regulates Wound Healing-associated Differentiation in Regenerating Human Skin Epithelium

**Figure 8:**
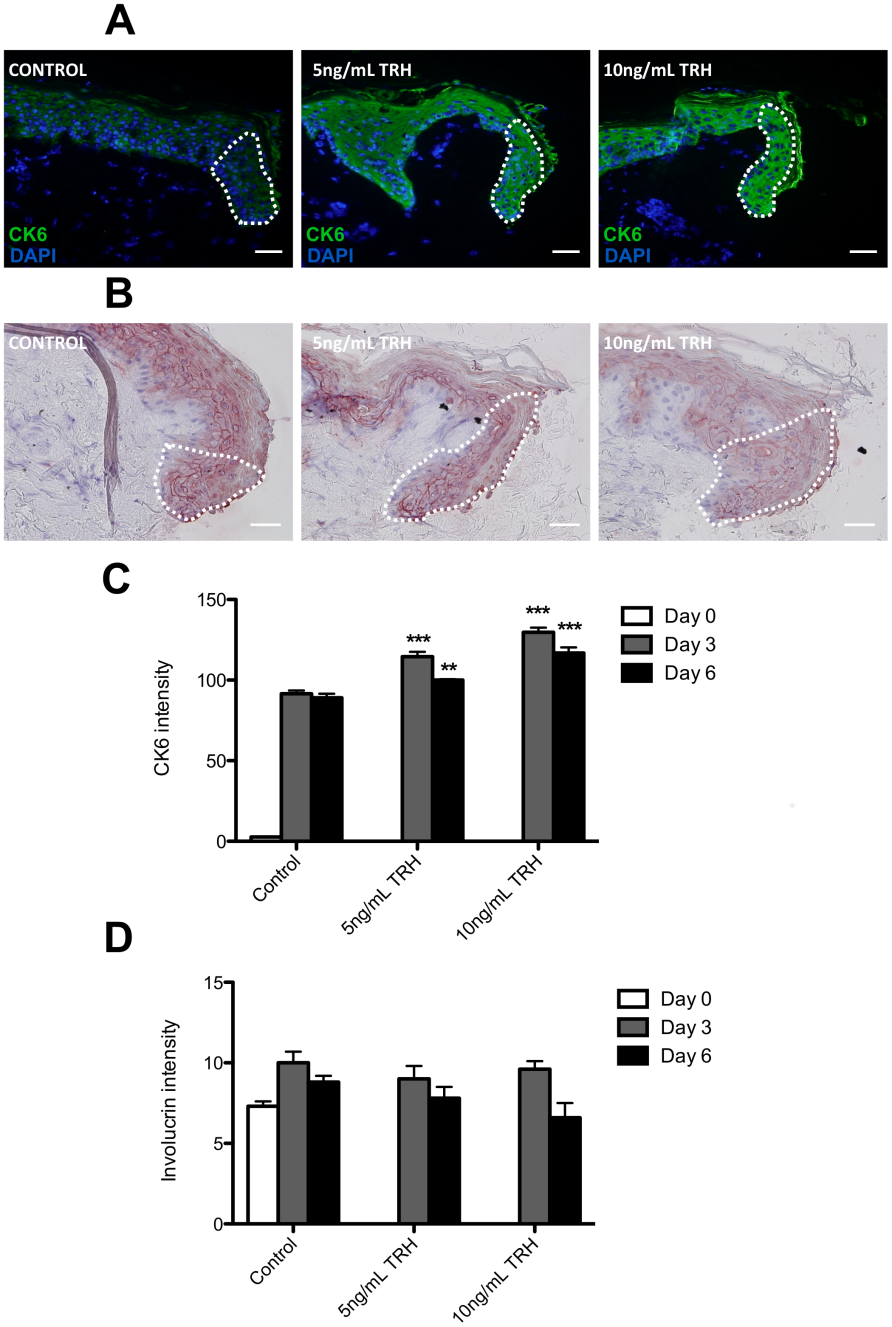
TRH increases cytokeratin 6 but not involucrin expression in human skin punch wounds. (**A**) Representative images indicated cytokeratin 6 (CK6) expression in control, 5 ng/mL and 10 ng/mL TRH-treated human skin punch wound sections, 6 Days post-wounding. The white-hatched line demarcates new epithelial tongue. (**B**) Representative images indicating involucrin (Inv) expression in control, 5 ng/mL and 10 ng/mL TRH-treated human skin punch wound sections, 6 Days post-wounding. The white-hatched line demarcates new epithelial tongue. (**C**) Quantification of involucrin immunoreactivity in the new epithelial tongues indicated in (A). (**D**) Quantification of CK6 immunoreactivity in the new epithelial tongues indicated in (B). Data are mean ± SEM of 4 female donors. Significance relative to control data at the same time-point denoted by **P<0.01, ***P<0.001.

In comparison to vehicle controls, TRH treatment significantly up-regulated protein expression of the wound healing-associated keratin, CK6 (Figures 8A,C). This correlated with significantly increased epithelial tongue migration as early as day 3 of organ culture (Figure 7). By contrast involucrin immunoreactivity was not significantly altered by TRH compared to vehicle controls (Figure 8B,D), suggesting that TRH does not greatly impact on keratinocyte terminal differentiation under assay conditions.

## Discussion

Utilising a comparative approach to investigating cutaneous wound healing, this study demonstrates for the first time that the side-by-side analysis of organ-cultured adult frog and human skin can provide an instructive and novel tool to investigate evolutionarily conserved, clinically relevant modulators of vertebrate wound healing. Recent literature on human skin explants for wound healing studies [29] has highlighted the utility of such techniques, in particular the advantages offered over simple *in vitro* techniques such as keratinocyte scratch assays [30], [31]. The comparative biology approach advocated here facilitates a reliable assessment of the potential for therapeutic agents to enhance sub-optimal wound healing, as found in chronic human skin ulcers. As such, it provides direct translational relevance.

Frog skin organ culture has been practised since the beginning of the 20th century [34] though mostly in tadpoles and froglets [35], [58]. Our data highlight the benefits of employing an adult frog skin *ex vivo* assay, specifically utilising the skin of *X. tropicalis*, so as to model and dissect conserved principles that also underlie adult human skin healing. As demonstrated by the wealth of literature dedicated to the study of wound healing and regeneration in amphibians [10]–[18], it is clear that these vertebrates provide a valuable model in which to study these processes, and insights from these lower vertebrate models may well be relevant to human wound healing. The adult frog skin assay reported here is intended to complement, rather than replace, well-established and instructive embryonic frog wound healing methodologies [59]–[61]. Indeed, we propose that adult frog skin will provide a powerful system to facilitate the translation of key findings from these embryonic/tadpole stage experiments into preclinical human skin research.

Utilising a combination of the historically well-developed approaches to frog skin organ culture [32], [34], [62] with more recent methodologies for studying *ex vivo* wound repair and the punch-in-a-punch design [29], [36], we demonstrate here that re-epithelialisation of wounded frog skin can be stimulated both with normal adult female frog serum and with the recognised wound-healing promoter, 17β-estradiol [39]. Employing a previously described technique for the serum-free organ culture of full-thickness human skin [37] and the punch-in-a-punch design [29], [36], these effects are mirrored in the epidermal regeneration of adult human skin in organ culture, which is also promoted by 17β-estradiol and human serum. In both assay systems, 17β-estradiol and species-specific serum can therefore be employed as physiologically relevant positive control agents.

Although the serum used in these investigations was largely uncharacterised, it is plausible that the wound-healing promoting effects of serum supplementation are determined, at least in part, by the presence of 17β-estradiol. Indeed, Hecker *et al* [63] measured serum estradiol levels from male *X. laevis* frogs to be in the region of 3 ng/ml (corresponding to approximately 12 nM). We tested the levels of both 17β-estradiol and TRH in the female frog serum used in this study, demonstrating a 17β-estradiol concentration almost 3 times greater than was reported [63] in male animals. As no trace of TRH could be detected in the serum we used, we suggest that 17β-estradiol is at least partially responsible for the wound-promoting effects of serum addition.

The stimulation of re-epithelialisation is likely the result of a combination of keratinocyte migration and increased proliferation. Indeed, whereas it is well recognised that epiboly [49] is an important early response to skin wounding *in vitro*, both frog and human skin explants also displayed increased proliferation within the newly formed epithelial tongues as early as 1 day post-wounding (Figures 2,3,4). Therefore, whilst epithelial migration will almost certainly play a role in this initial re-epithelialisation, it appears that keratinocyte proliferation is also stimulated early in the repair process.

Since adult frog skin, in particular with respect to wound healing, has only been sparsely investigated, few well-characterized antibodies are commercially available for frog skin immunohistology. We thus purposely employed readily available histological and morphometric techniques, and demonstrate that these simple markers suffice to quantitatively answer basic wound healing questions such as the extent of re-epithelialisation or epithelial proliferation/apoptosis. Given that the high cutaneous concentration of TRH in frog skin is well established [20], our finding raises the possibility that one of the, as yet poorly understood functions of intracutaneous TRH may be to facilitate epithelial regeneration after wounding. As our data has shown that wounded frog skin, cultured in the absence of serum, may also display spontaneous re-epithelialisation, this raises the question of whether TRH, produced and retained within the explants might be responsible for this. Further experimentation aimed at inhibiting TRH function in this system is required to address this possibility.

TRH, administered at concentrations that we had previously shown to stimulate human hair growth and mitochondrial energy metabolism of human epidermis and hair follicles *in vitro* [22]–[24], also promotes the re-epithelialisation of adult wounded human skin. On the one hand, this demonstrates that the wound healing-stimulatory properties of TRH have been conserved over a wide expanse of vertebrate evolution. On the other, it underscores the similarities and cross-connections between wound healing and hair growth and also suggests that known hair growth-promoting agents also deserve to be explored in a wound-healing context [27], [28].

Clinically, TRH is a particularly interesting candidate wound healing promoter. It is an unusually stable, well-tested tripeptide that has long been administered systemically in daily endocrinological practice (i.e.,TRH stimulation test for thyroid function) and as such is a relatively inexpensive treatment [64]. This greatly facilitates drug repositioning and subsequent testing of TRH in clinical wound healing trials.

TRH exerts its effects via the TRH receptor, of which 3 *Xenopus* subtypes have been reported in *X. laevis* [65], [66], designated *trhr1*, *trhr2* and *trhr3*. This contrasts to mammalian species in which only 2 TRH receptor isoforms have been identified. Differential TRH responses may therefore be accounted for through signalling via specific *trhr* isoforms. In examining the tissue distribution of the *trhr* isoforms in *X. laevis*, Bidaud *et al* [65], [66] identified *trhr1* in the dorsal skin. While that study was performed in *X. laevis*, the high degree of conservation of gene expression patterns in *X. laevis* and *X. tropicalis* [67] makes it very likely that the same expression profile for these genes will be evident in *X. tropicalis*. Indeed, the *X. tropicalis* genome contains orthologs of these three *trhr* genes, previously identified in *X. laevis* [68].

Normal human skin and/or its appendages express both TRH and TSH as well as their cognate receptors [19], [22], [42], [43], [69], and TRH stimulates TSH expression in human epidermis [43]. Interestingly, TSH up-regulates the transcription of connective tissue growth factor, a key promoter of wound healing [9], [70] in human skin appendages [42], and stimulates mitochondrial biogenesis and energy metabolism in normal human epidermis [71]. Thus, besides the documented direct stimulatory effects of TRH on human keratinocyte proliferation [22] and mitochondrial function [23] *in situ*, the re-epithelialisation-promoting effects of TRH seen in wounded frog and human skin *in vitro* may reflect, in part, the stimulation of intracutaneous TSH production.

The mechanism(s) through which TRH acts in the frog/human skin remain(s) to be fully elucidated. TRH binding to a TRH GPCR isoform would trigger a signalling cascade involving phospholipase C, an increase in downstream inositol 1,4,5-trisphosphate (InsP_3_) production and activation of protein kinase C. Intriguingly, a recent report using *Xenopus* embryonic wound healing assays has shown that both InsP_3_ and its metabolite, inositol 1,3,4,5-tetrakisphosphate (InsP_4_), are able to accelerate the speed of wound healing [72]. Further signalling events triggered by TRH binding can involve PKC-dependent or independent activation of mitogen-activated protein kinase (MAPK). However, it is not clear whether these signalling events occur in human/frog skin during wounding, in response to TRH. Indeed, TRH-induced phosphorylation of epidermal growth factor receptor enhances the MAPK cascade, and as such it is possible that receptor cross-regulation is involved in modulating integrated signalling pathways. Indeed, as TRH receptor mutations have not been reported to cause skin abnormalities [73], it is certainly plausible that TRH may exert its wound healing-promoting effects via TRHR-independent mechanisms [23]. The precise mechanism of TRH-signalling in both Xenopus and human skin therefore deserves further investigation.

An additional mechanism may be the TRH-mediated stimulation of CK6 expression, a wound-healing associated keratin [52], [74]. Knocking-out CK6 in mice delays re-epithelialisation, despite apparently normal keratinocyte proliferation [74], yet paradoxically, reports also indicate that CK6 itself may reduce keratinocyte migration [52]. Interestingly, these newly identified CK6-regulatory effects of TRH seem to differ between human hair follicle keratinocytes and regenerating wounded human epidermis. Microarray analysis had suggested that TRH reduces CK6 transcription in human hair follicle keratinocytes *in situ* [22]. In vehicle-treated wounded human skin punches we also saw a decline of CK6 mRNA levels after 11 days of culture (unpublished observation). Yet, CK6 protein-associated immunoreactivity was up-regulated by TRH early on. This suggests that CK6 is differentially regulated by TRH in human outer root sheath keratinocytes and wounded human epidermis.

One observation from this study is the lack of a clear concentration dependence in the response to TRH. Although only 2 concentrations were assessed in both frog and human systems, no obvious concentration-mediated changes were seen. This begs the question whether it is a short ‘spike’ in TRH concentration that is required to elicit downstream effects, rather than sustained levels. It is recognised that TRHRs may be rapidly internalized after TRH binding, thus acting as an agonist-induced desensitization mechanism [75]. As such, higher concentrations of TRH may lead to an increase in internalization, reducing the availability of receptors through which TRH can signal.

In conclusion, we have introduced TRH as a novel, evolutionarily conserved, neuroendocrine promoter of epidermal regeneration that deserves further clinical exploration. In addition we advocate a comparative biology approach to wound healing research and report two simple, complementary *adult* frog and human skin *ex vivo*-assays that facilitate the identification and standardized preclinical testing of clinically relevant stimulators of wound healing.

## Supporting Information

Figure S1 **TRH has no impact on dermal proliferation in** ***X. tropicalis*** **skin explants.** (**A–D**) Representative images of PH3 immunoreactivity in *X. tropicalis* skin explants after 7 days culture with the indicated treatments. White dotted line demarcates the *Stratum spongiosum* and the *Stratum compactum* (see Figure 1). (**E**) The graph displays the percentage of PH3+ cells identified by analysis of 3 high-powered fields per section, with 3 sections per animal analysed. (**F**) The graph shows the percentage of mitotic figures identified by Weigert’s staining in the *X. tropicalis* dermis. 200 nuclei were analysed per skin section, with 3 sections per animal counted. Data are mean ± SEM of 4 frogs (2 male and 2 female).
(PNG)
