# Supplementary figures and images for: Thyrotropin-Releasing Hormone (TRH) Promotes Wound Re-Epithelialisation in Frog and Human Skin
Source: PLoS One. 2013 Sep 2;8(9):e73596. doi: 10.1371/journal.pone.0073596 (PMC3759422; doi:10.1371/journal.pone.0073596)

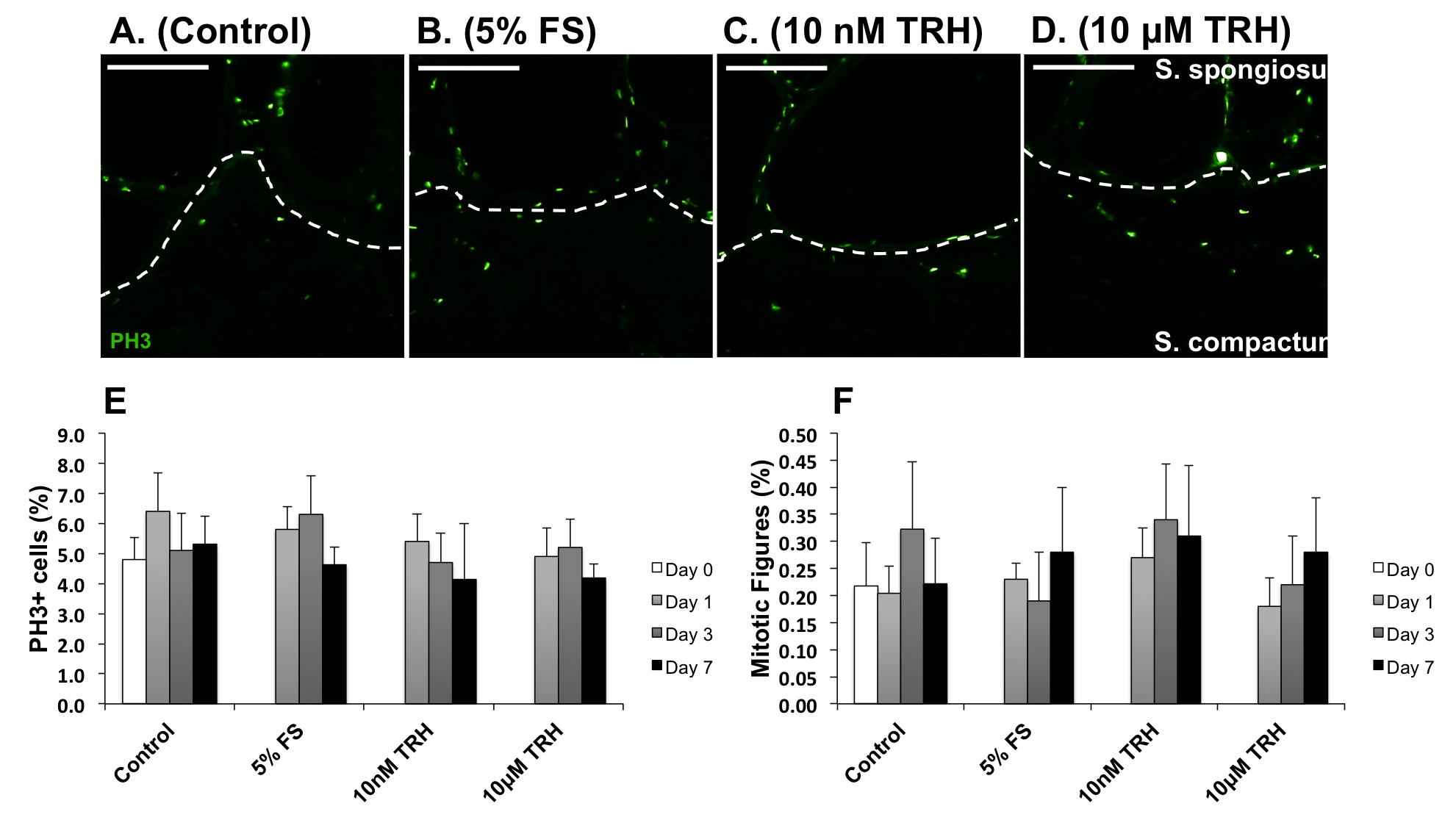

Supplement: Figure S1 — TRH has no impact on dermal proliferation in X. tropicalis skin explants. (A–D) Representative images of PH3 immunoreactivity in X. tropicalis skin explants after 7 days culture with the indicated treatments. White dotted line demarcates the Stratum spongiosum and the Stratum compactum (see Figure 1). (E) The graph displays the percentage of PH3+ cells identified by analysis of 3 high-powered fields per section, with 3 sections per animal analysed. (F) The graph shows the percentage of mitotic figures identified by Weigert’s staining in the X. tropicalis dermis. 200 nuclei were analysed per skin section, with 3 sections per animal counted. Data are mean ± SEM of 4 frogs (2 male and 2 female). (PNG) [file pone.0073596.s001.png]
